# Supplementary material for: Engineering a passivating electric double layer for high performance lithium metal batteries
Source: Nat Commun. 2022 Apr 19;13:2029. doi: 10.1038/s41467-022-29761-z (PMC9018679; doi:10.1038/s41467-022-29761-z)
Supplement: Supplementary file 1 — Supplementary Information [file 41467_2022_29761_MOESM1_ESM.pdf]

Engineering a passivating electric double layer for high performance lithium metal batteries

Weili Zhang[1], Yang Lu[1], Lei Wan[1], Pan Zhou[1], Yingchun Xia[1], Shuaishuai Yan[1], Xiaoxia Chen[1], Hangyu Zhou[1], Hao Dong[1], Kai Liu*[1]

[1]Department of Chemical Engineering, Tsinghua University, China

*Corresponding author. E-mail: liukai2019@tsinghua.edu.cn

Table of Contents

Supplementary Fig. 1. ^{7}Li NMR spectra of LiFSI/DME with 0.3 M different additives.

Supplementary Fig. 2. Formation cycle of Li||NMC811 cells using LiFSI/DME and LiFSI-$LiNO_3$/DME at 0.2 C rate.

Supplementary Fig. 3. Long-term cycling performance of Li||NMC811 cells using LiFSI/DME and LiFSI-$LiNO_3$/DME at 0.1 C rate.

Supplementary Fig. 4. Long-term cycling performance of Li||NMC811 cells using LiFSI/DME and LiFSI-$LiNO_3$/DME at 1.0 C rate.

Supplementary Fig. 5. Long-term cycling performance of Li||NMC811 cells using LiFSI/DME and LiFSI-$LiNO_3$/DME at 3.0 C rate.

Supplementary Fig. 6. Long-term cycling performance of Li||NMC811 cells using LiFSI/DME and LiFSI-$LiNO_3$/DME at 5.0 C rate.

Supplementary Fig. 7. Long-term cycling performance of Li||NMC811 cells using LiFSI/DME and LiFSI-$LiNO_3$/DME at 10.0 C rate.

Supplementary Fig. 8. Long-term cycling performance of Li||NMC811 cells using LiFSI/DME and LiFSI-$LiNO_3$/DME at 5.0 C charge and 1.0 C discharge.

Supplementary Fig. 9. Long-term cycling performance of Li||NMC811 cells using different electrolytes.

Supplementary Fig. 10. Cross-sectional FIB/SEM images of NMC811 particles after 100 cycles in Li||NMC811 cells with 1 M LiFSI/DME and LiFSI-$LiNO_3$/DME.

Supplementary Fig. 11. Voltage curves of the lithium metal anodes (right axis) and NMC811 cathodes (Left axis) with respect to the lithium foil reference electrodes during charge and discharge at 0.2 C, 0.5 C, 1.0 C, 3.0 C and 5.0 C current density.

Supplementary Fig. 12. Li metal performance of the LiFSI-$LiNO_3$/DME.

Supplementary Fig. 13. Verify universality with alternative additives and solvents.

Supplementary Fig. 14. Discharge profiles and cycling performance of Li||NMC811 cells using conventional electrolytes $LiPF_6$/EC-DEC and LiFSI-$LiNO_3$/DME at different temperatures. Cycling performance of Li||NMC811 cells in LiFSI-$LiNO_3$/THF electrolyte at -40 °C and 0.5 C rate.

Supplementary Fig. 15. 3-Electrode impedance study of NMC811||Li||Li cells at 25 °C and -20 °C.

Supplementary Fig. 16. Kinetics of interfacial processes at the cathode/electrolyte interface measured by EIS using a 3-electrode setup.

Supplementary Fig. 17. Conductivity versus temperature of different electrolytes.

Supplementary Fig. 18. Comparison of relevant low-temperature LMBs as quantified by Supplementary Table 3.

Supplementary Fig. 19. Voltage profiles of the Li||NMC811 cell using 1.0 M LiFSI/DME after cycled 10 cycles in LiFSI-$LiNO_3$/DME.

Supplementary Fig. 20. Voltage profiles of the Li||NMC811 cell using 1.3 M LiFSI/DME.

Supplementary Fig. 21. Voltage profiles of the Li||NMC811 cell using LiFSI-$LiBF_4$/DME.

Supplementary Fig. 22. The number density profiles of bound-DME

Supplementary Fig. 23. Local structure evolution of inner-Helmholtz interfacial regions at cathode surface in 1 M LiFSI/DME under different voltage.

Supplementary Fig. 24. Local structure evolution of inner-Helmholtz interfacial regions at cathode surface in 1 M LiFSI-$LiNO_3$/DME under different voltage.

Supplementary Fig. 25. Collected clusters from inner-Helmholtz interfacial regions in LiFSI-$LiNO_3$/DME system.

Supplementary Fig. 26. Comparison of number densities of pure solvent (DME) obtained from MD and AIMD simulation at 0V.

Supplementary Fig. 27. Comparison of orientation of the DME adsorbed at the electrode obtained from MD and AIMD simulation.

Supplementary Fig. 28. Comparison of number densities of the bound-DME in the interfacial region at different electrolytes system.

Supplementary Fig. 29. Local structure evolution of inner-Helmholtz interfacial regions at cathode surface in 1 M LiFSI-$LiNO_3$/DME under different voltage.

Supplementary Fig. 30. Snapshots of inner-Helmholtz interfacial regions of the cathode surface in 1 M LiFSI- Li_2SO_4/DME at different voltage.

Supplementary Fig. 31. Photos of the liquid cup, a key part of the sealed AFM electrochemical cell.

Supplementary Fig. 32. Cyclic voltammograms of LiFSI/DME and LiFSI-$LiNO_3$/DME using

stainless steel working electrode in a 2032-coin cell setup.

Supplementary Fig. 33. SEM images of (a) pristine stainless steel electrode and stainless steel electrode after Cyclic voltammograms test in (b) LiFSI/DME (c) LiFSI-$LiNO_3$/DME

Supplementary Table 1. The volume of the various anions calculated by DFT.

Supplementary Table 2. Li^+ transference number (t_{Li+}) computed from DC polarization measurements at 10 mV using the Bruce–Vincent method.

Supplementary Table 3. Metrics of interest for previously published low temperature Li metal batteries.

Supplementary Notes

Supplementary References

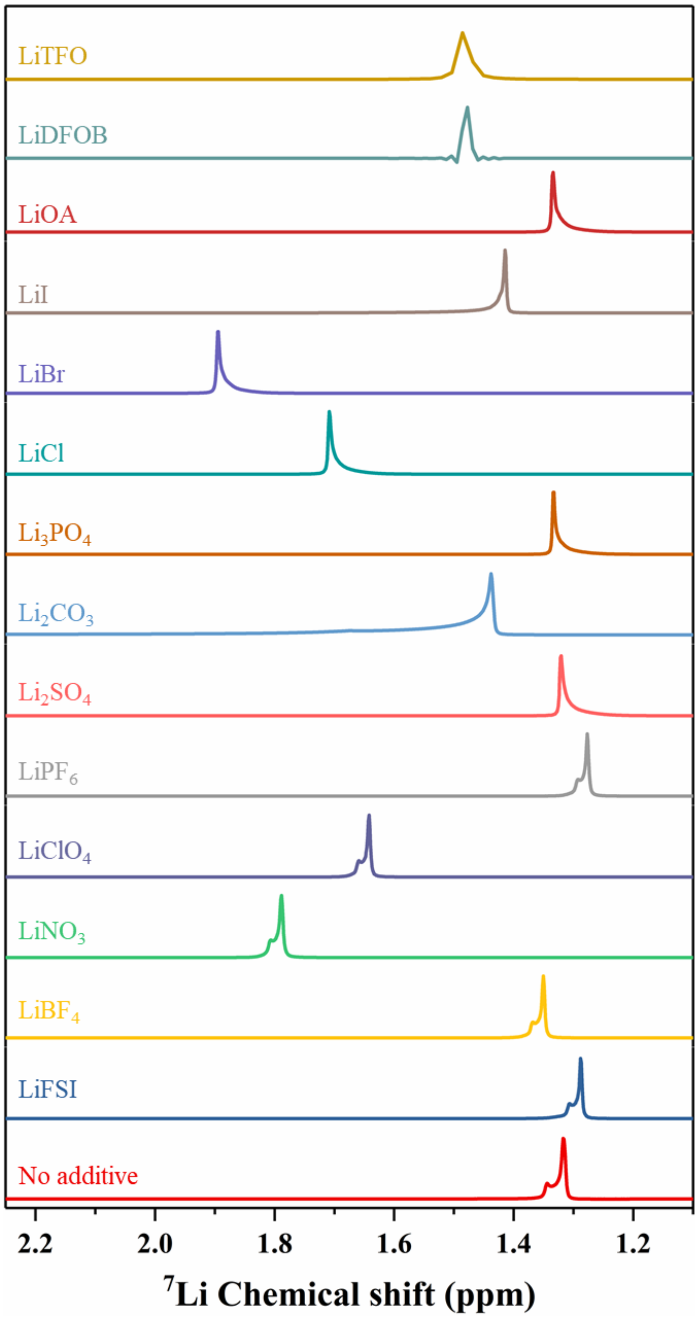

Supplementary Fig. 1. ^{7}Li NMR spectra of LiFSI/DME with 0.3 M different additives. It should be noted that the solubility of some additives such as Li_2CO_3, Li_2SO_4, Li_3PO_4, etc., is less than 0.3 M, which is characterized by saturation state.

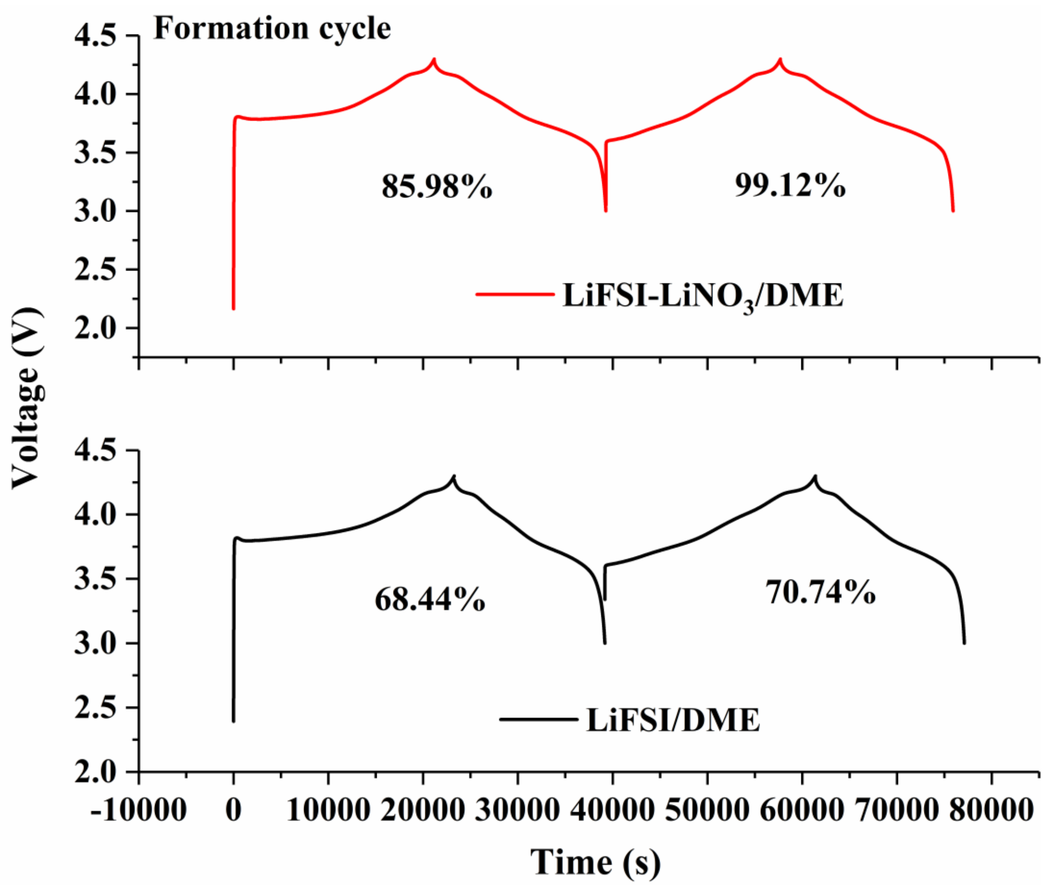

Supplementary Fig. 2. Formation cycle of Li||NMC811 cells using LiFSI/DME and LiFSI-$LiNO_3$/DME at 0.2 C rate.

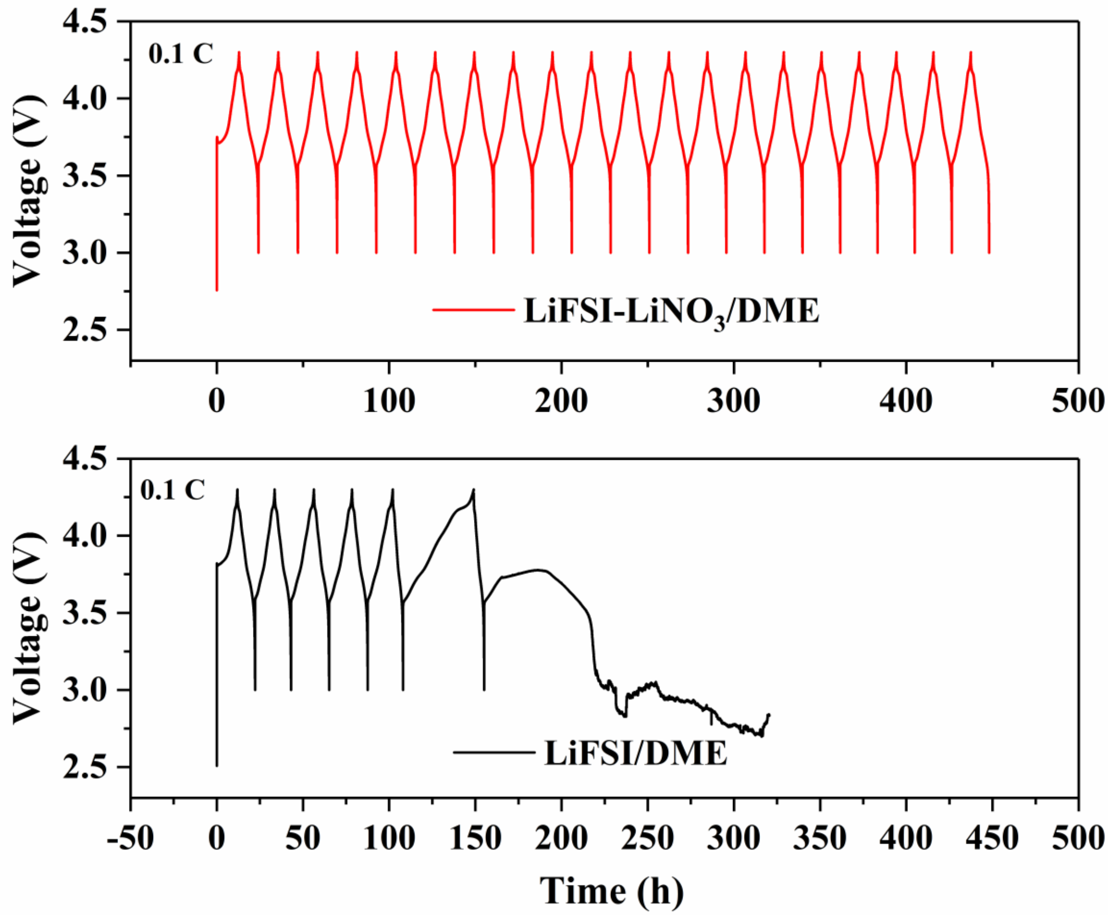

Supplementary Fig. 3. Long-term cycling performance of Li||NMC811 cells using LiFSI/DME and LiFSI-$LiNO_3$/DME at 0.1 C rate.

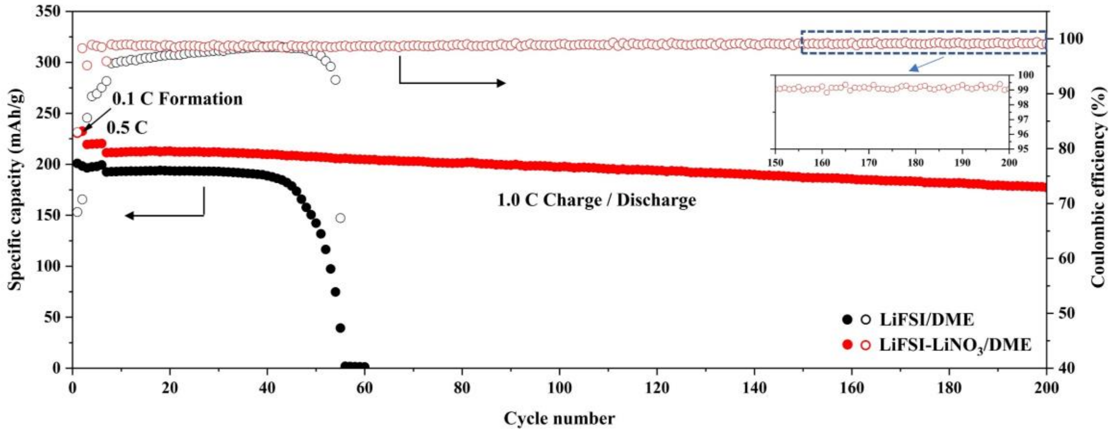

Supplementary Fig. 4. Long-term cycling performance of Li||NMC811 cells using LiFSI/DME and LiFSI-LiNO$_3$/DME at 1.0 C rate.

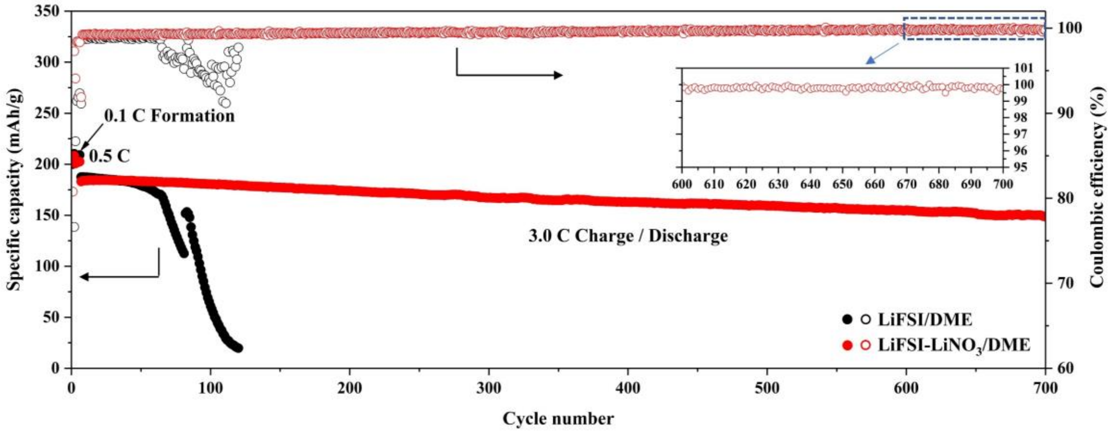

Supplementary Fig. 5. Long-term cycling performance of Li||NMC811 cells using LiFSI/DME and LiFSI-LiNO$_3$/DME at 3.0 C rate.

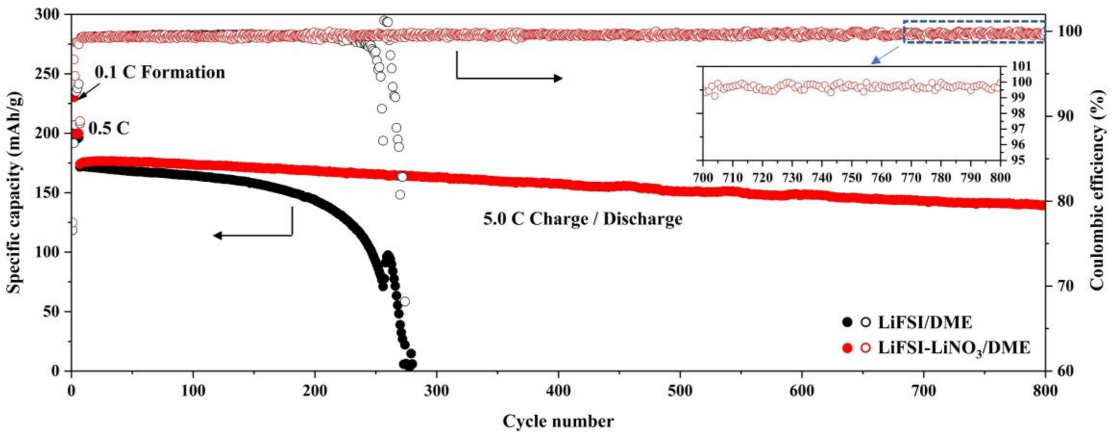

Supplementary Fig. 6. Long-term cycling performance of Li||NMC811 cells using LiFSI/DME and LiFSI-$LiNO_3$/DME at 5.0 C rate.

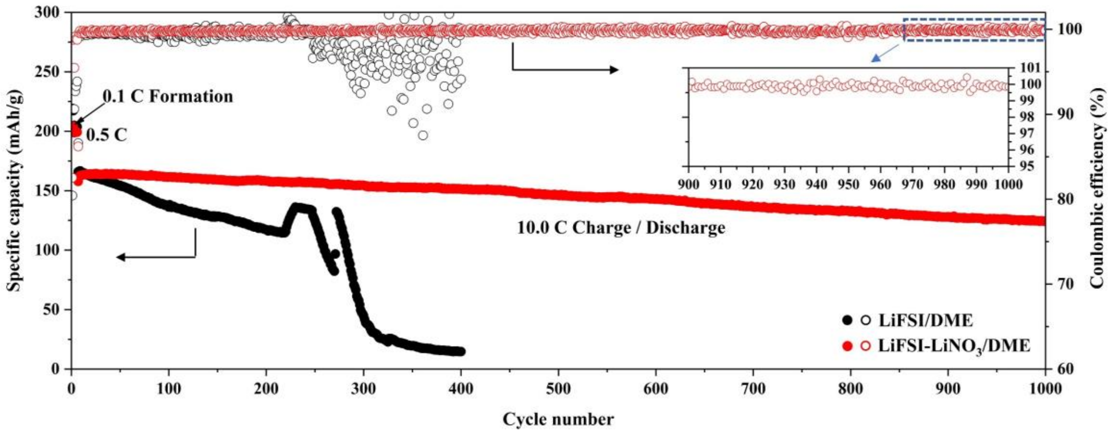

Supplementary Fig. 7. Long-term cycling performance of Li||NMC811 cells using LiFSI/DME and LiFSI-$LiNO_3$/DME at 10.0 C rate.

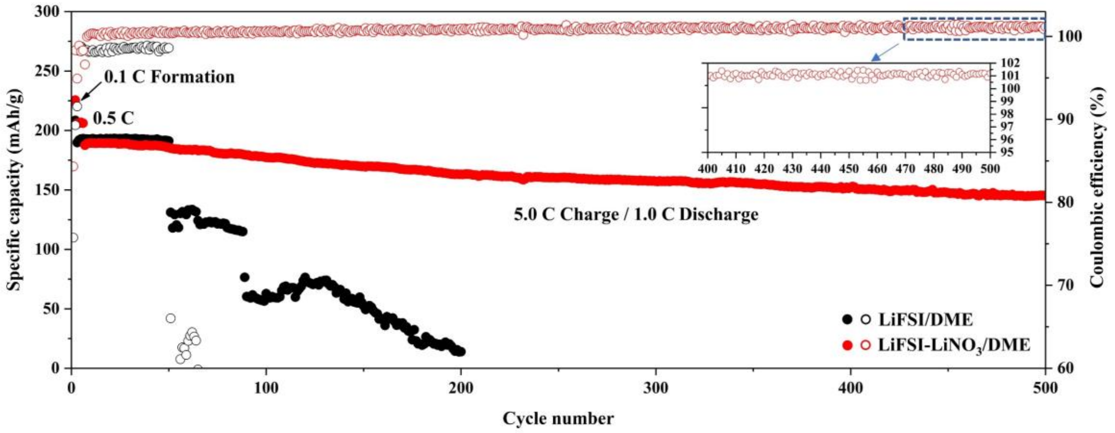

Supplementary Fig. 8. Long-term cycling performance of Li||NMC811 cells using LiFSI/DME and LiFSI-$LiNO_3$/DME at 5.0 C charge and 1.0 C discharge.

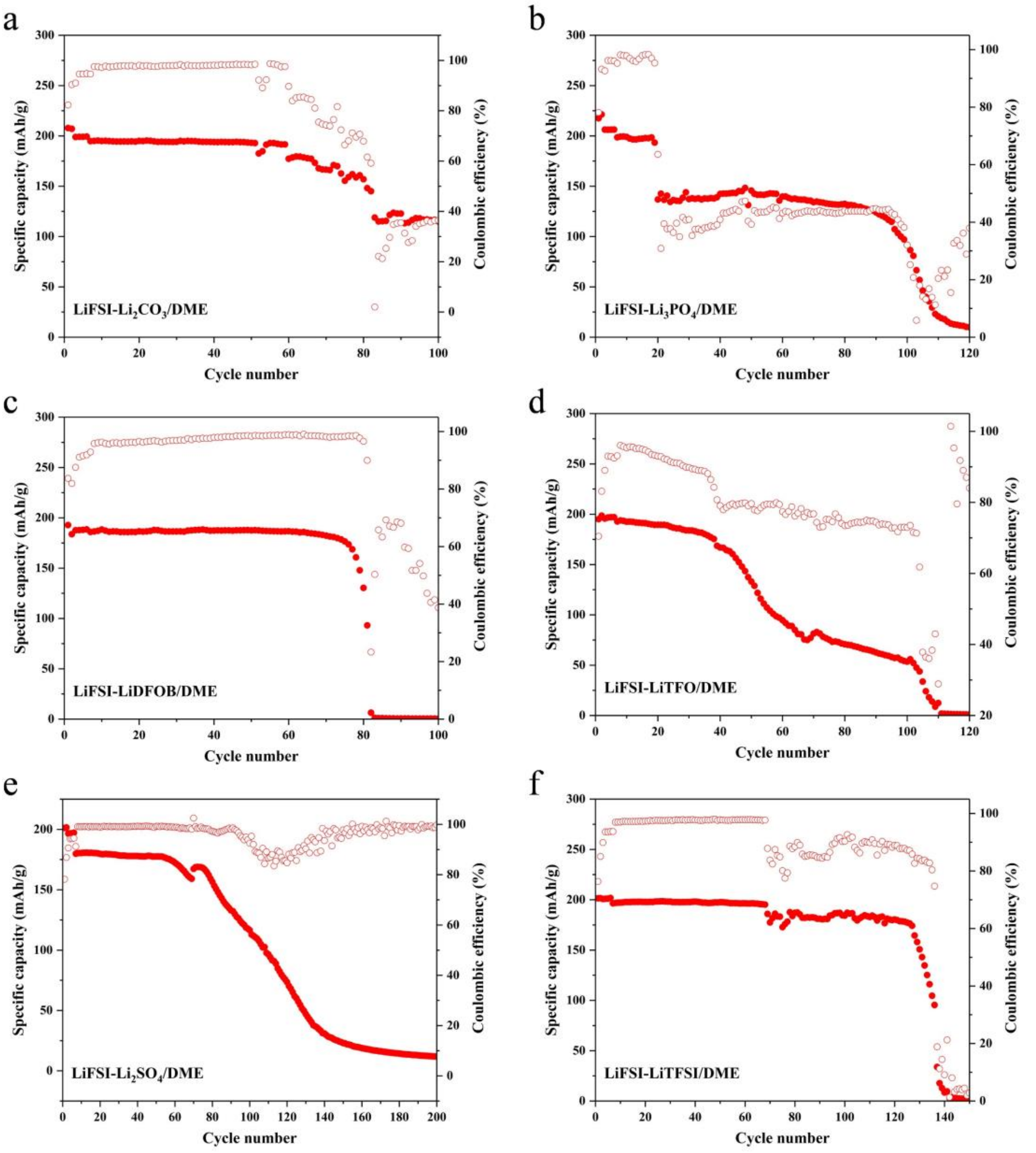

Supplementary Fig. 9. Long-term cycling performance of Li||NMC811 cells using (a) LiFSI-Li_2CO_3/DME, (b) LiFSI-Li_3PO_4/DME, (c) LiFSI-LiDFOB/DME, (d) LiFSI-LiTFO/DME, (e) LiFSI-Li_2SO_4/DME and (f) LiFSI-LiTFSI/DME at 1.0 C rate.

a b

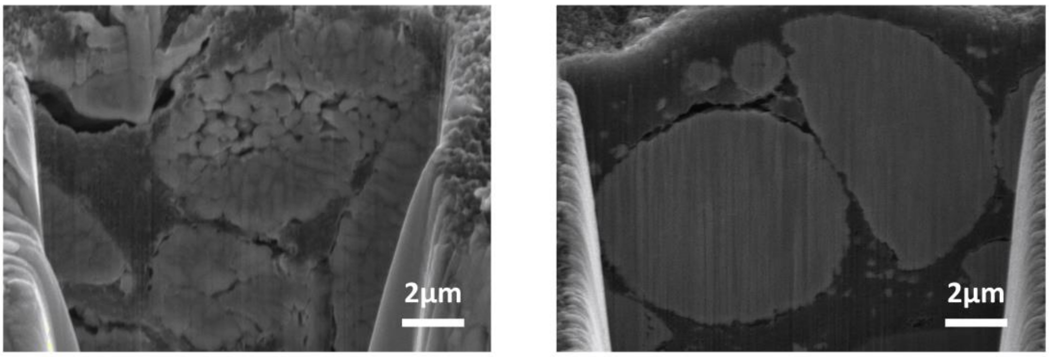

Supplementary Fig. 10. Cross-sectional FIB/SEM images of NMC811 particles after 100 cycles in Li||NMC811 cells with (a) 1 M LiFSI/DME and (b) LiFSI-$LiNO_3$/DME.

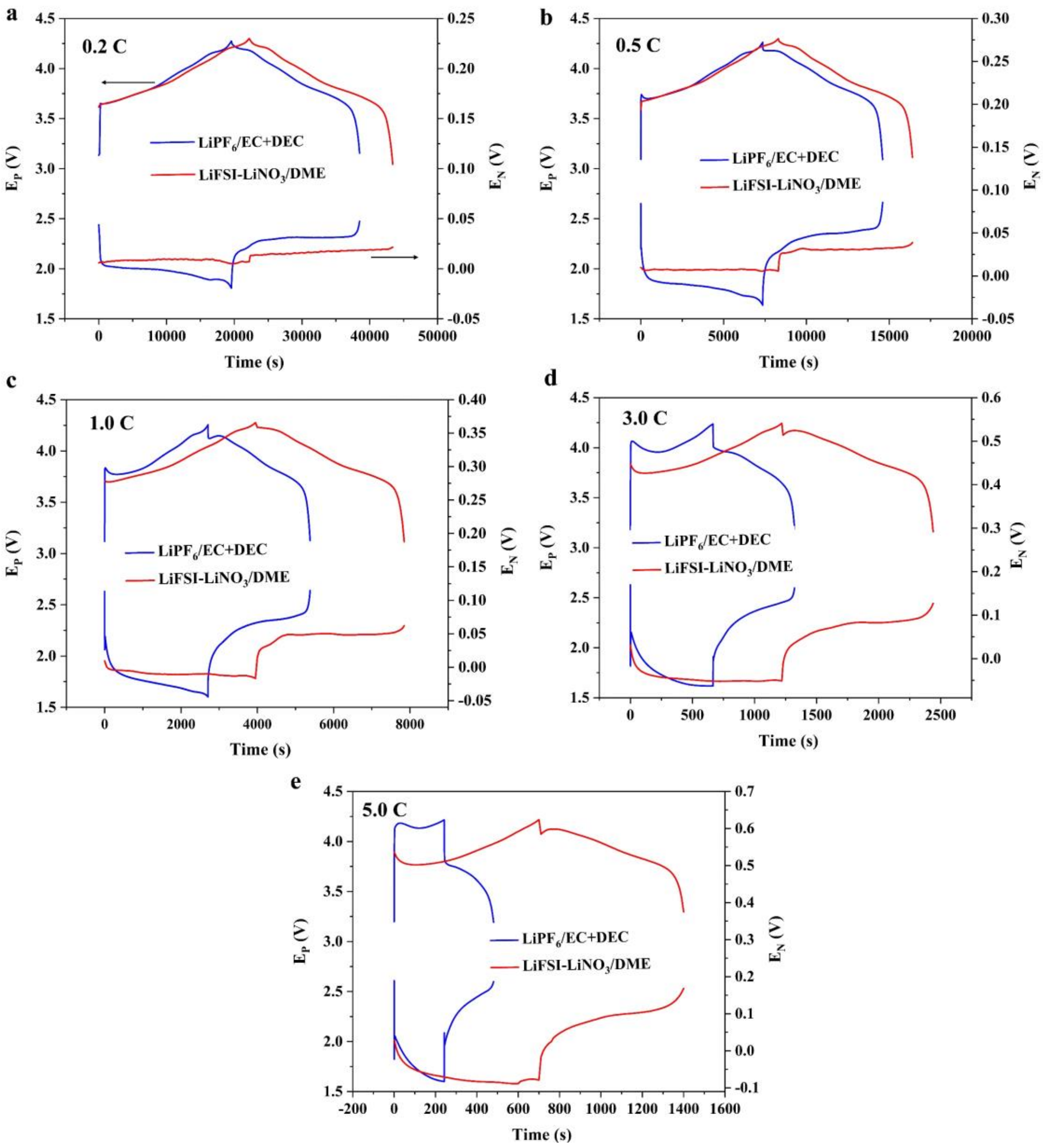

Supplementary Fig. 11. Voltage curves of the lithium metal anodes (right axis) and NMC811 cathodes (Left axis) with respect to the lithium foil reference electrodes during charge and discharge at (a) 0.2 C (b) 0.5 C (c) 1.0 C (d) 3.0 C (e) 5.0 C current density.

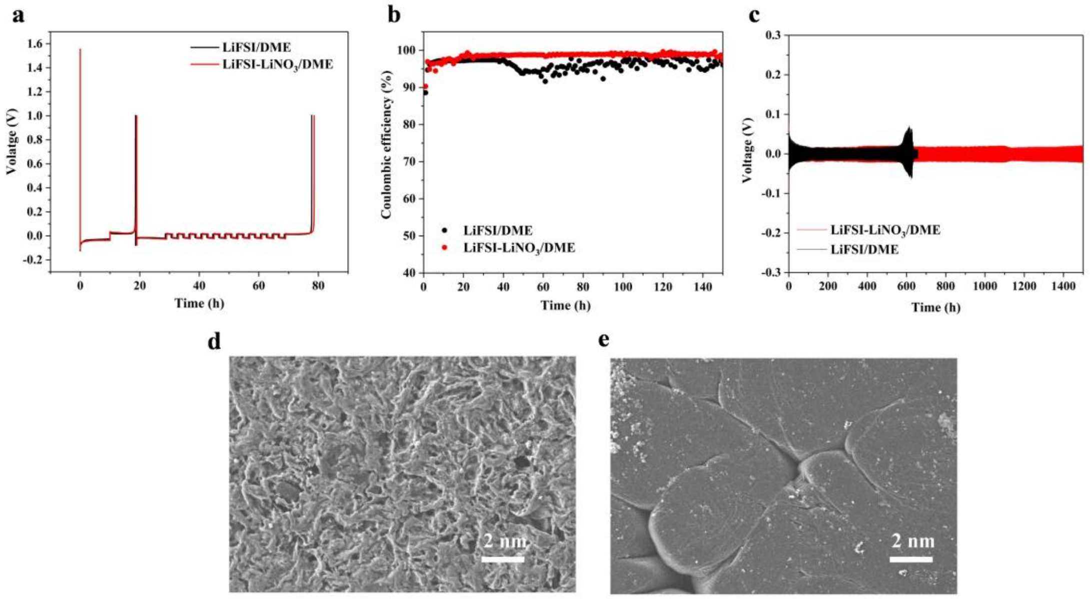

Supplementary Fig. 12. Li metal performance of the LiFSI-$LiNO_3$/DME. (a) Aurbach CE [32] of Li||Cu cells. (b) Cycling CE of Li||Cu cells. (c) Cycling performance of Li||Li cells. Li plating morphology on copper working electrodes in the (d) electrolyte without $LiNO_3$ and (e) the electrolyte with $LiNO_3$. The current density is 1 mA cm^{-2} and the plating capacity is 1 mAh cm^{-2}.

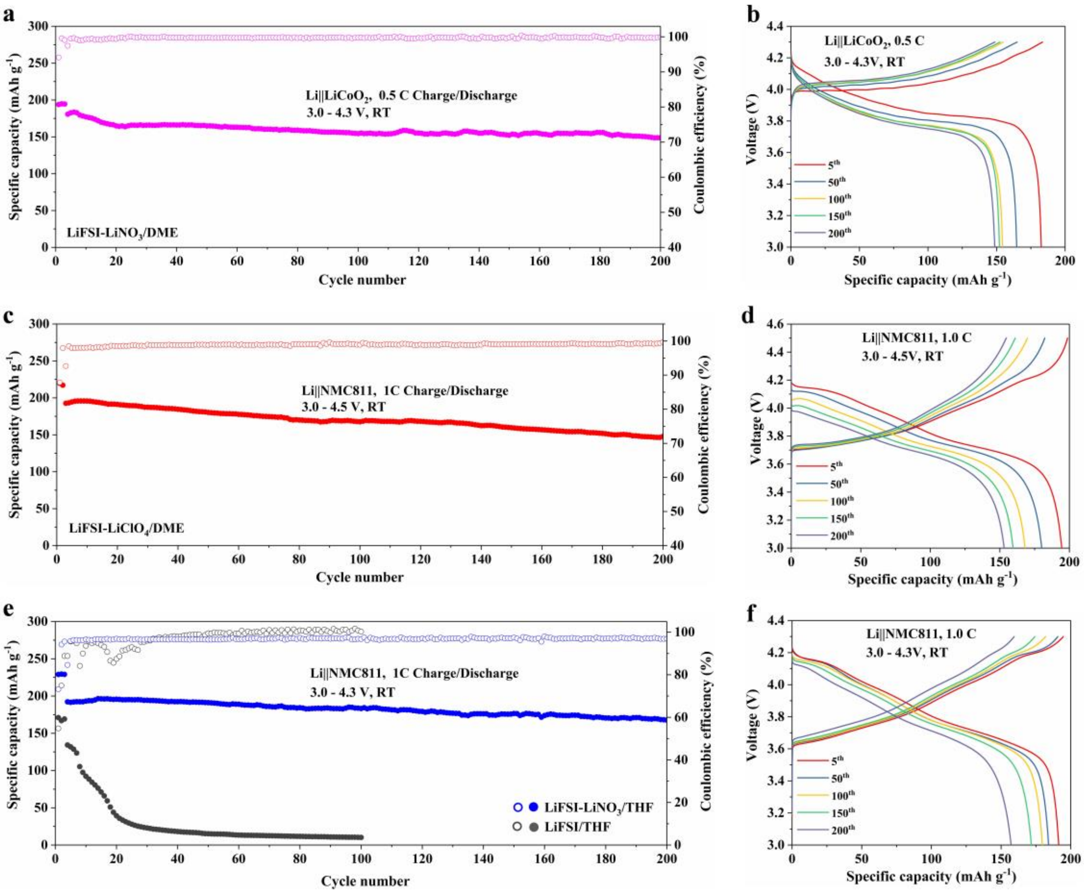

Supplementary Fig. 13. Verify universality with alternative additives and solvents. (a) Long-term cycling performances and (b) the corresponding voltage profiles at different cycles of high-voltage Li||$LiCoO_2$ cell using LiFSI-$LiNO_3$/DME at 0.5 C rate (voltage range: 3.0-4.3 V). (c) Long-term cycling performances and (d) the corresponding voltage profiles at different cycles of high-voltage Li||NMC811 cell using LiFSI-$LiClO_4$/DME at 1.0 C rate (voltage range: 3.0-4.5 V). (e) Cycling performance of Li||NMC811 cells using LiFSI-$LiNO_3$/THF and LiFSI/THF at 1.0 C rate (voltage range: 3.0-4.3 V). (f) The corresponding voltage profiles at different cycles of Li||NMC811 cells using LiFSI-$LiNO_3$/THF at 1.0 C rate.

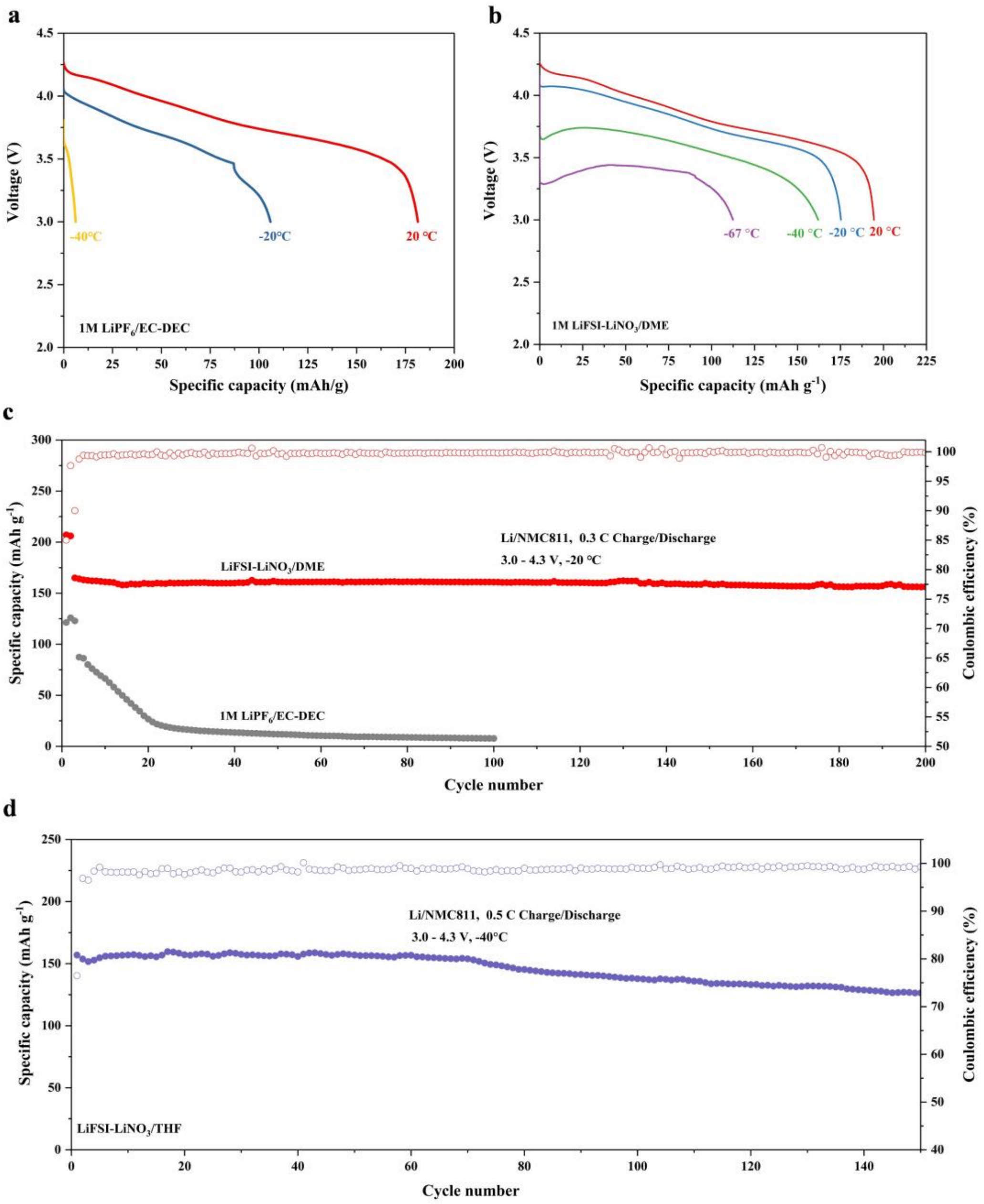

Supplementary Fig. 14. Discharge profiles of Li||NMC811 cells using (a) conventional electrolytes $LiPF_6$/EC-DEC, (b) LiFSI-$LiNO_3$/DME at different temperatures. (c) Cycling performance of Li||NMC811 cells in conventional $LiPF_6$/EC-DEC and LiFSI-$LiNO_3$/DME electrolyte at -20 °C and 0.3 C rate (d) Cycling performance of Li||NMC811 cells in LiFSI-$LiNO_3$/THF electrolyte at -40 °C and 0.5 C rate.

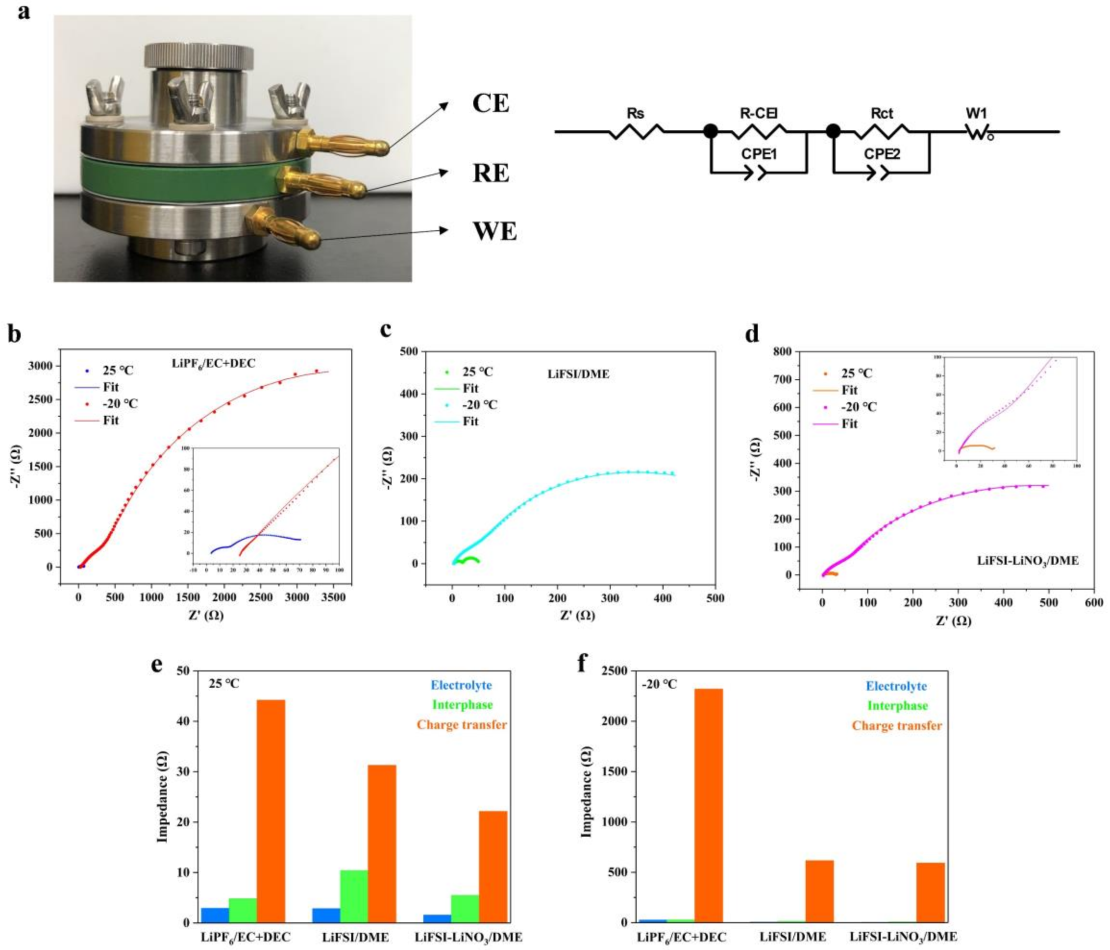

Supplementary Fig. 15. 3-Electrode impedance study of NMC811||Li||Li cells at 25 °C and -20 °C. a) Working schematic of the 3-electrode cell and Equivalent circuit model. Impedance spectra of the cathode at 50% state-of-charge (SOC) in b) $LiPF_6$/EC+DEC, c) LiFSI/DME and d) LiFSI-$LiNO_3$/DME. Breakdown of equivalent circuit elements from fit at (e) 25 °C and (f) -20 °C.

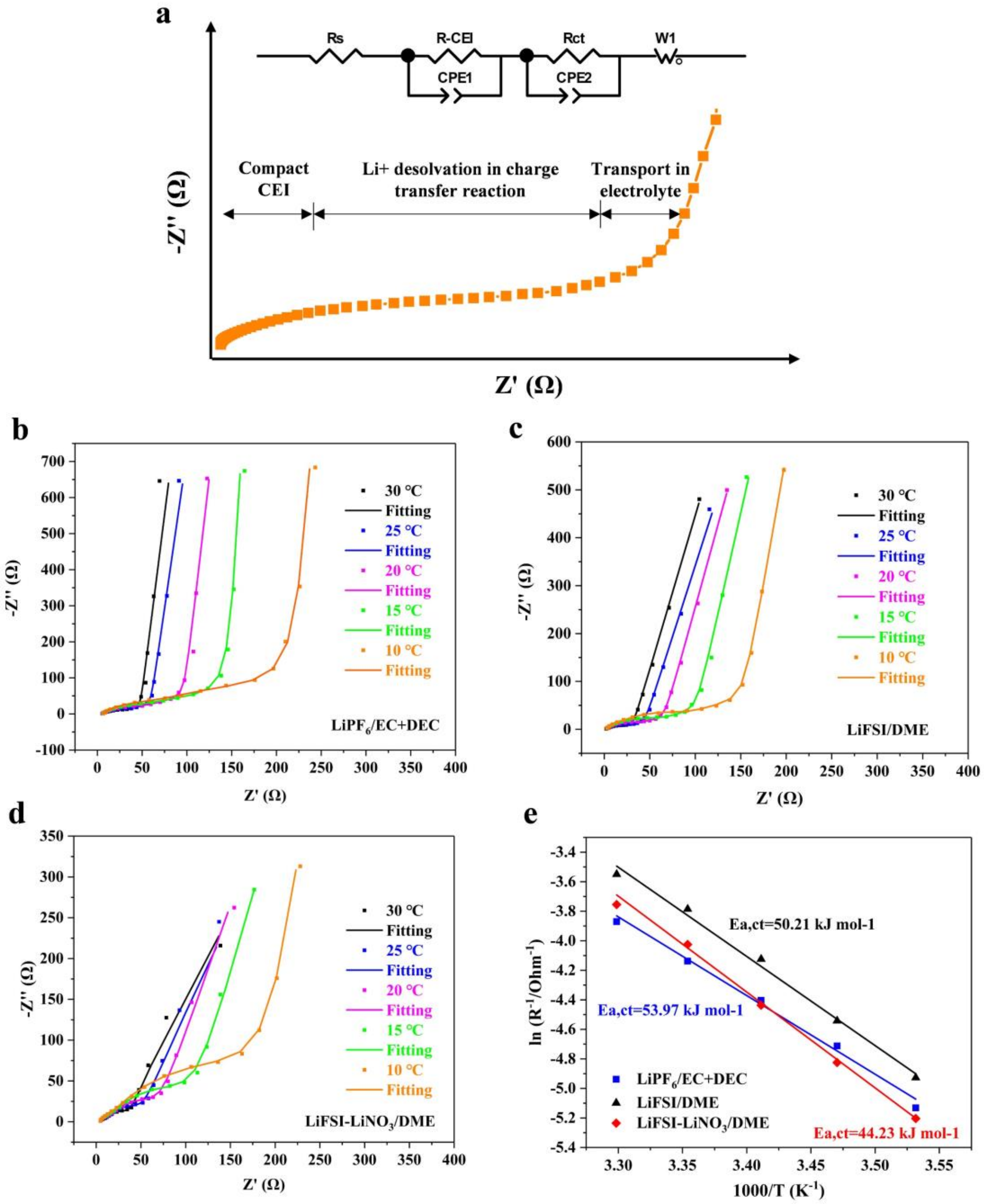

Supplementary Fig. 16. Kinetics of interfacial processes at the cathode/electrolyte interface measured by EIS using a 3-electrode setup. (a) equivalent circuit for EIS fitting and corresponding region in the Nyquist plot. Temperature dependent EIS curves of cells containing (b) $LiPF_6$/EC+DEC (c) LiFSI/DME and (d) LiFSI-$LiNO_3$/DME. (e) Arrhenius behavior of the resistance.

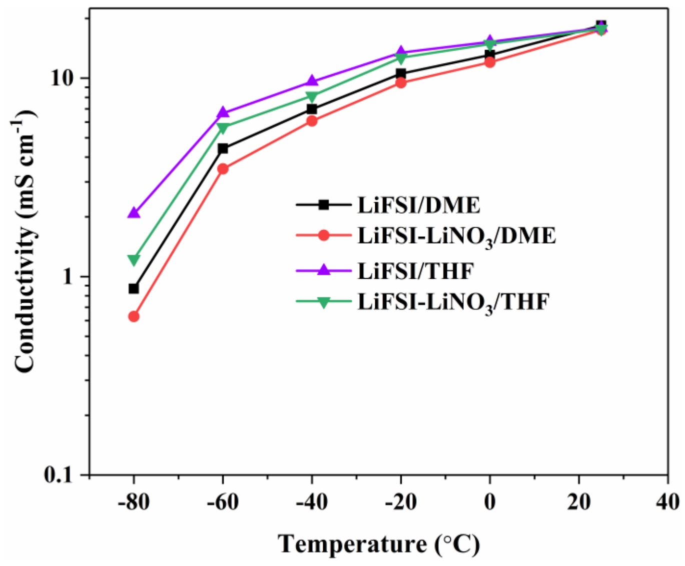

Supplementary Fig. 17. Conductivity versus temperature of different electrolytes.

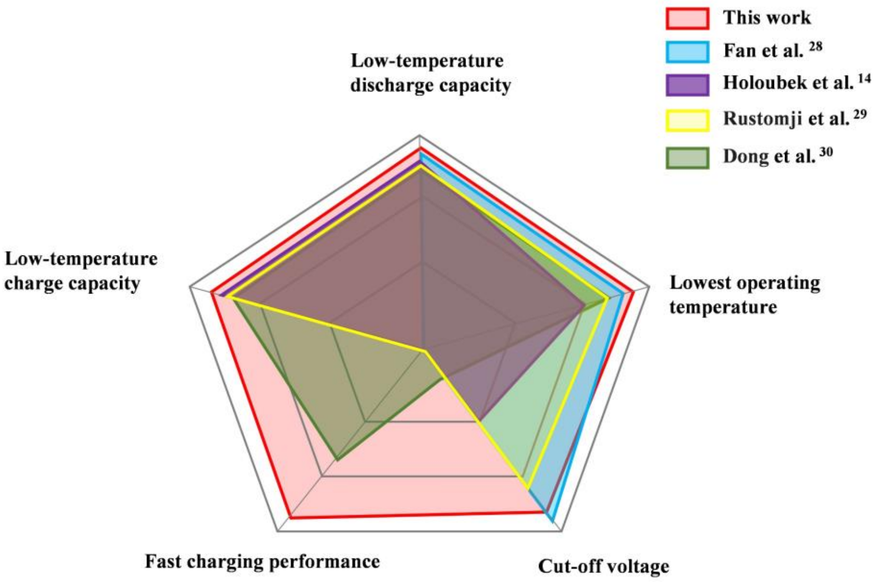

Supplementary Fig. 18. Comparison of relevant low-temperature LMBs as quantified by Supplementary Table 3.

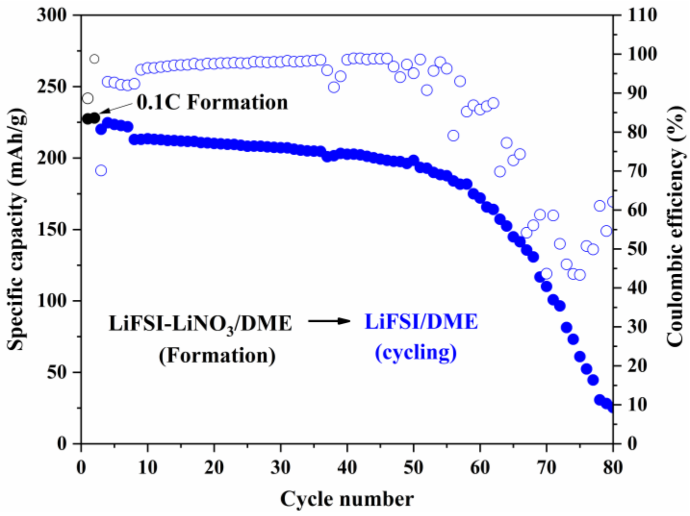

Supplementary Fig. 19. Voltage profiles of the Li||NMC811 cell using 1.0 M LiFSI/DME after cycled 10 cycles in LiFSI-LiNO$_3$/DME. The disassembled NMC811 cathode, Li anode and separator were washed with DME solvent for several times to remove residual lithium salt.

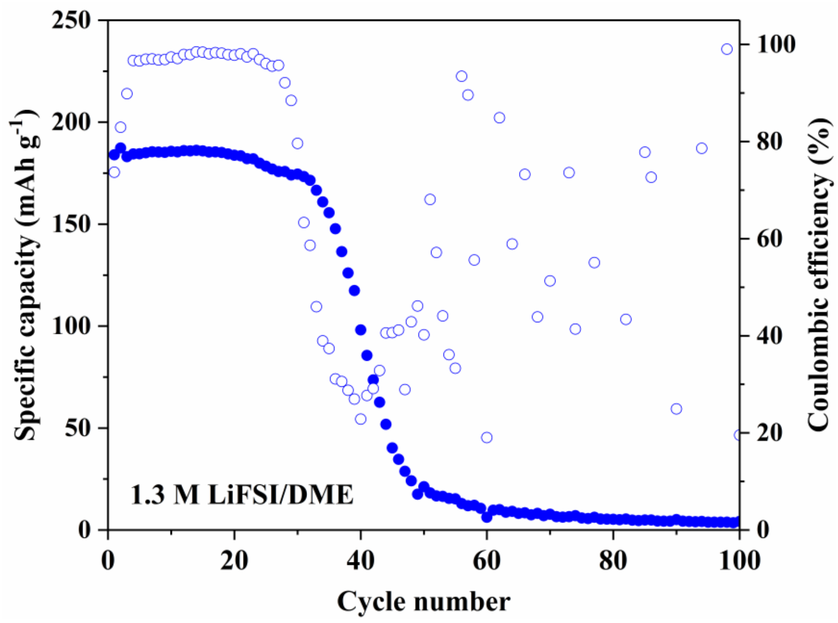

Supplementary Fig. 20. Voltage profiles of the Li||NMC811 cell using 1.3 M LiFSI/DME.

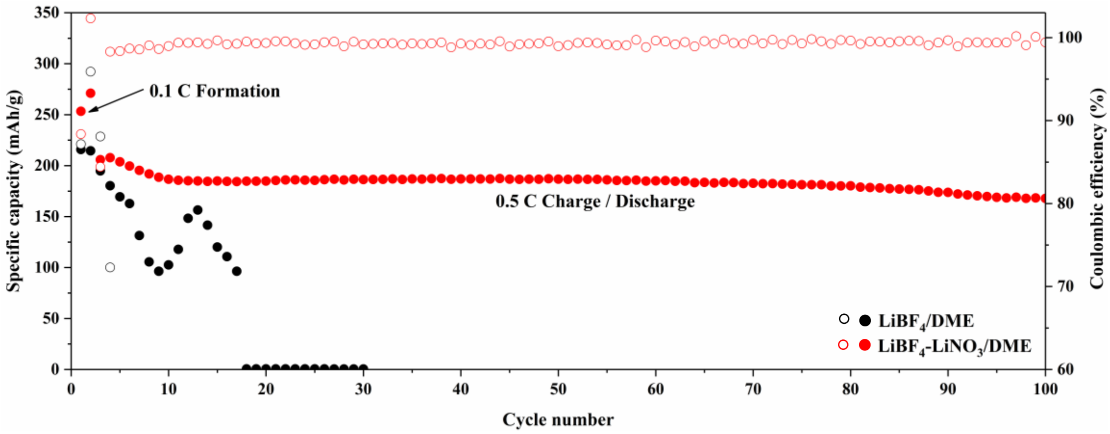

Supplementary Fig. 21. Voltage profiles of the Li||NMC811 cell using LiFSI-$LiBF_4$/DME.

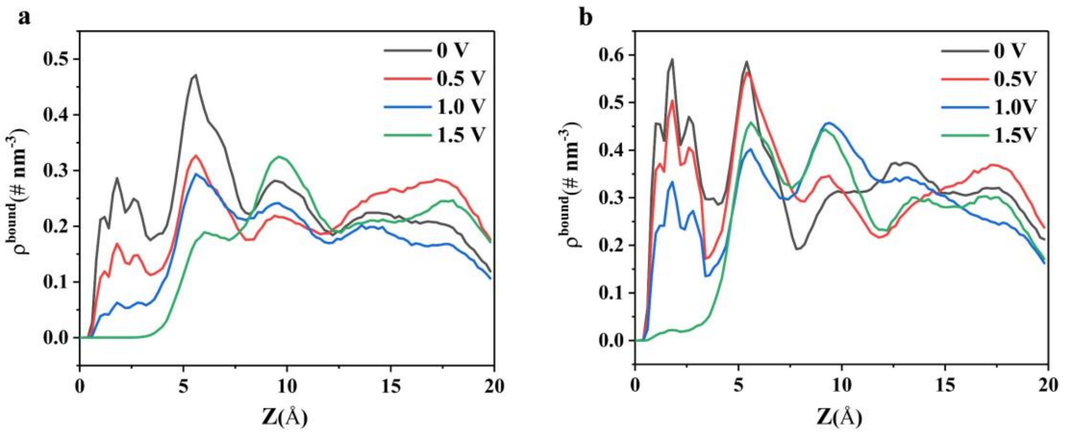

Supplementary Fig. 22. The number density profiles of bound-DME in (**a**) 1 M LiFSI/DME and (**b**) LiFSI-$LiNO_3$/DME as a function of distance from the electrode surface (z).

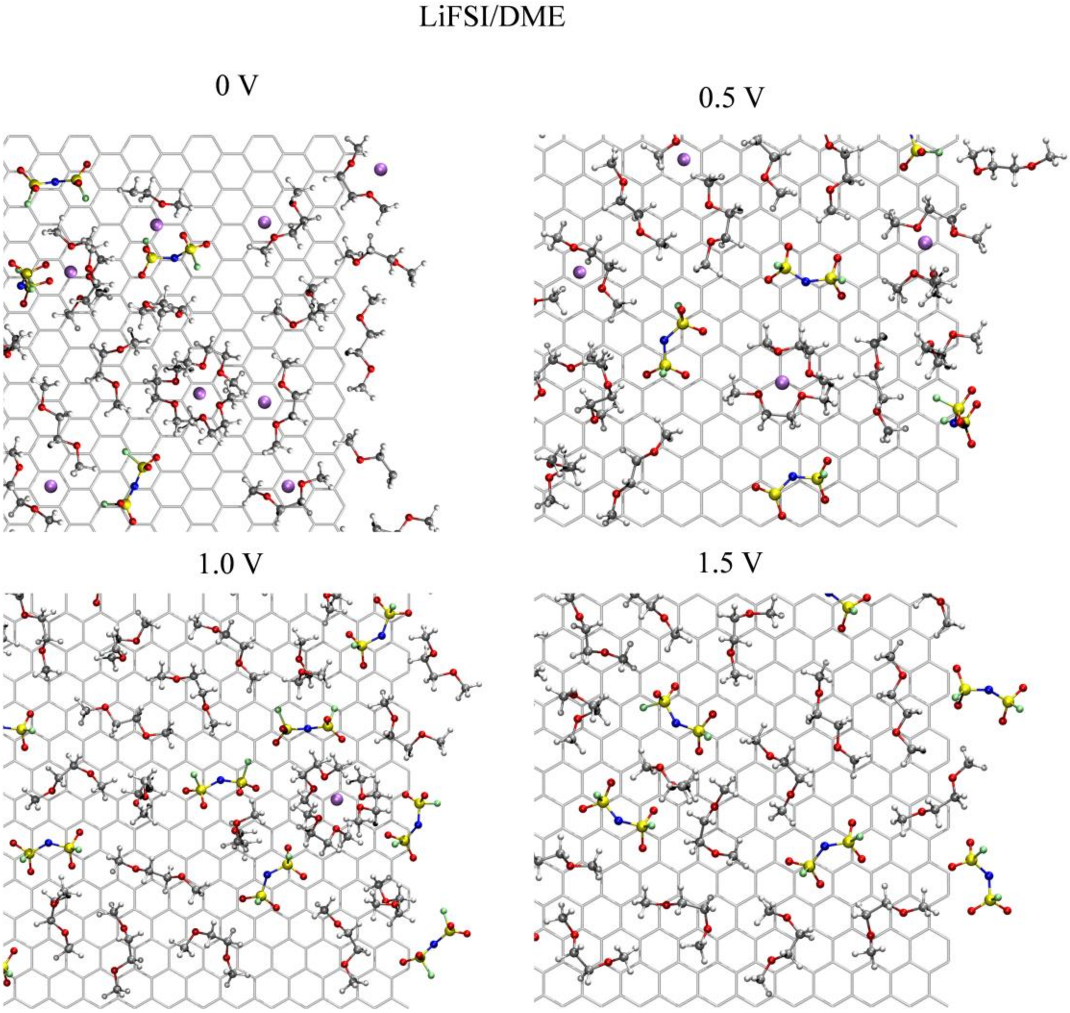

Supplementary Fig. 23. Local structure evolution of inner-Helmholtz interfacial regions at cathode surface in 1 M LiFSI/DME under different voltage.

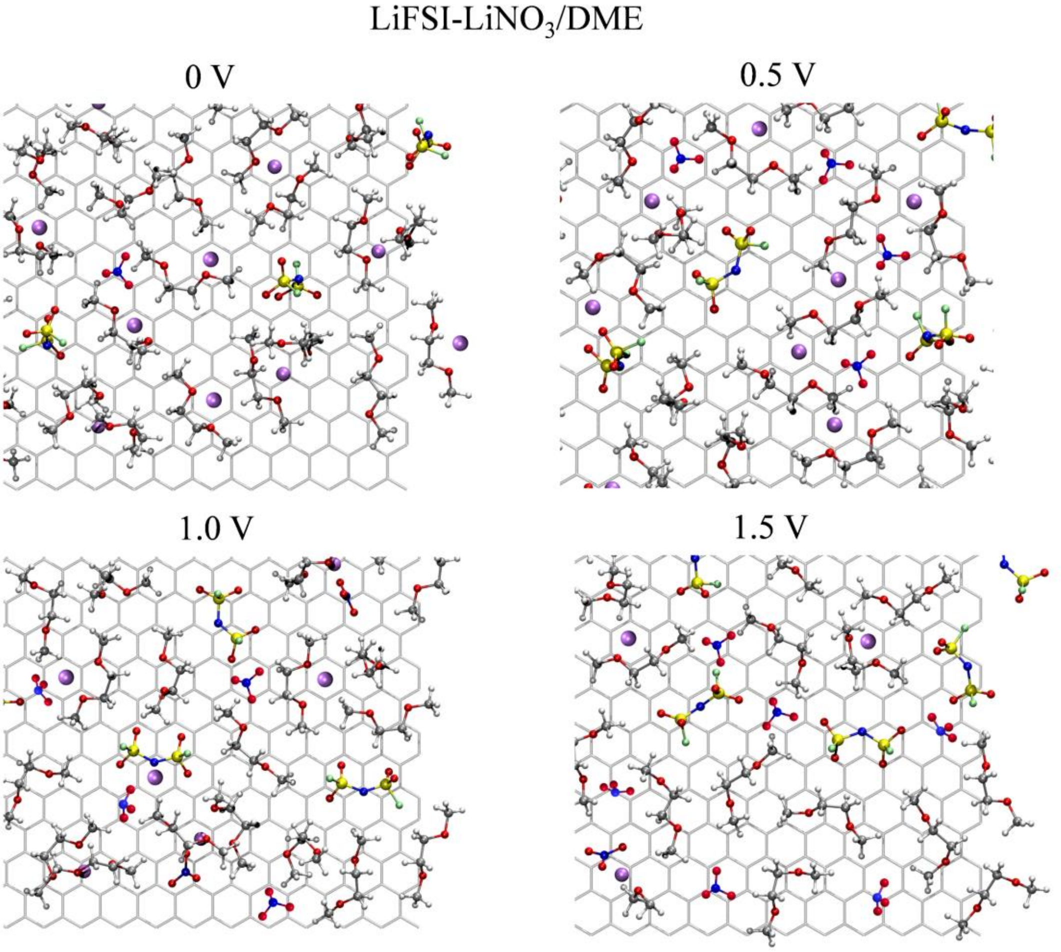

Supplementary Fig. 24. Local structure evolution of inner-Helmholtz interfacial regions at cathode surface in 1 M LiFSI-$LiNO_3$/DME under different voltage.

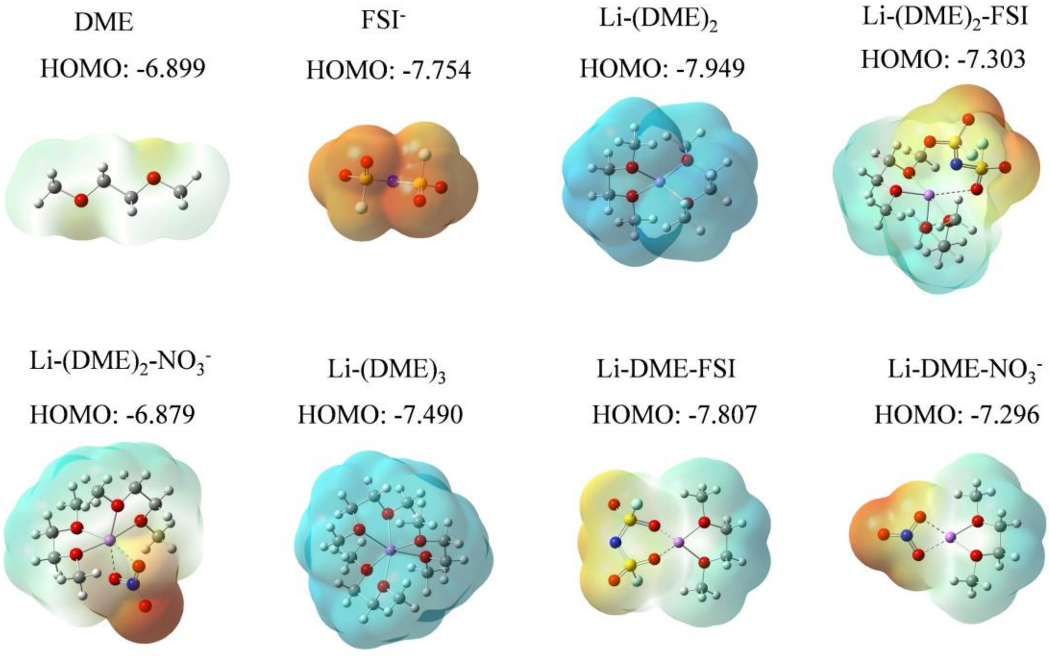

Supplementary Fig. 25. Collected clusters from inner-Helmholtz interfacial regions in LiFSI-$LiNO_3$/DME system.

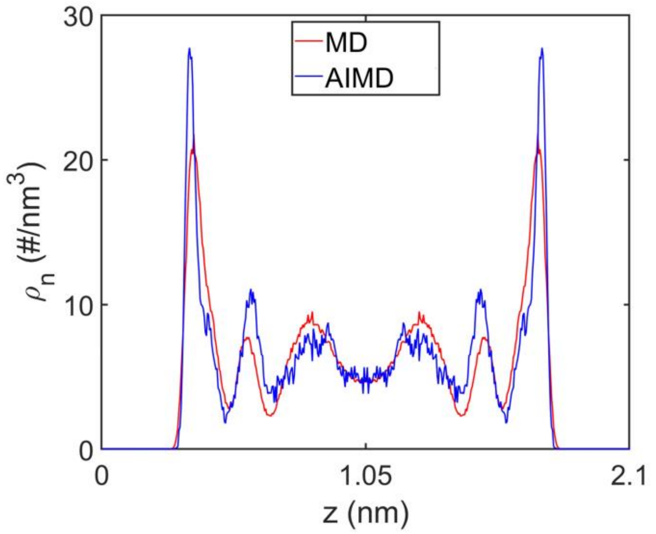

Supplementary Fig. 26. Comparison of number densities of pure solvent (DME) obtained from MD and AIMD simulation at 0V.

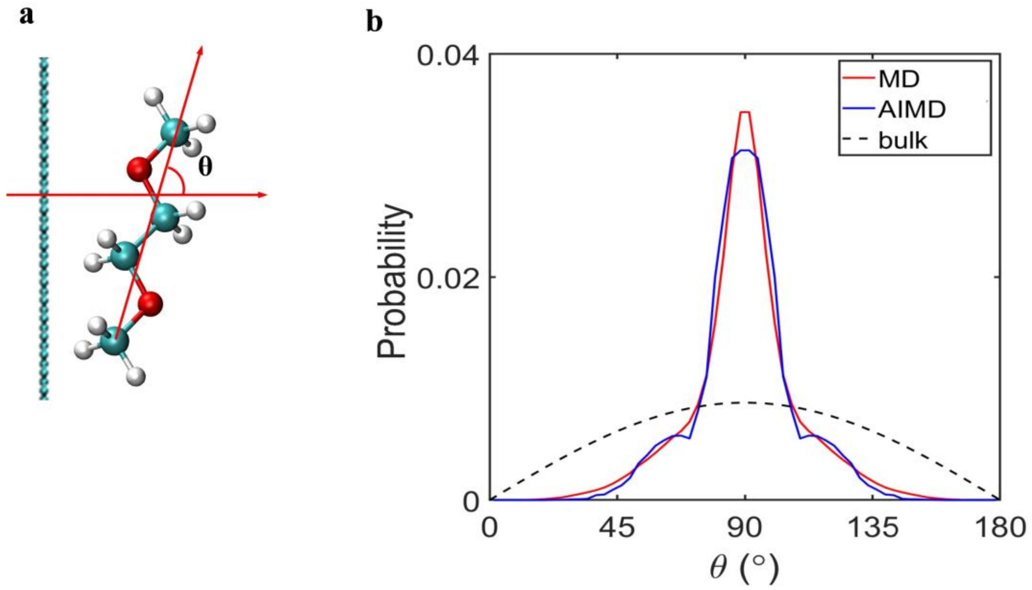

Supplementary Fig. 27. Comparison of orientation of the DME adsorbed at the electrode obtained from MD and AIMD simulation. (a) Schematics for cation orientation. (b) Probability distribution of θ with time

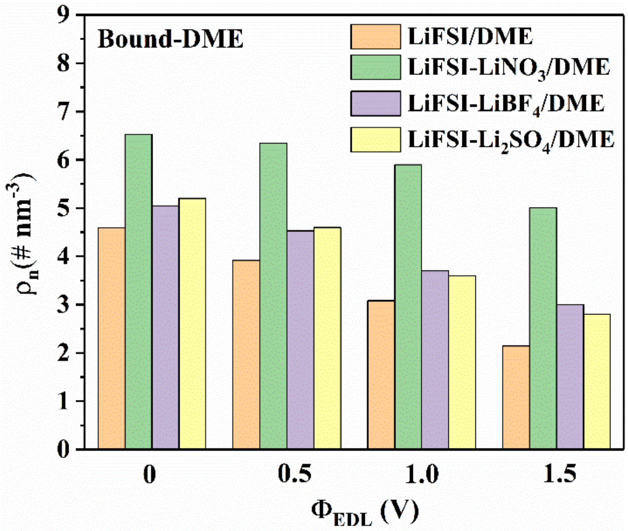

Supplementary Fig. 28. Comparison of number densities of the bound-DME in the interfacial region at different electrolytes system.

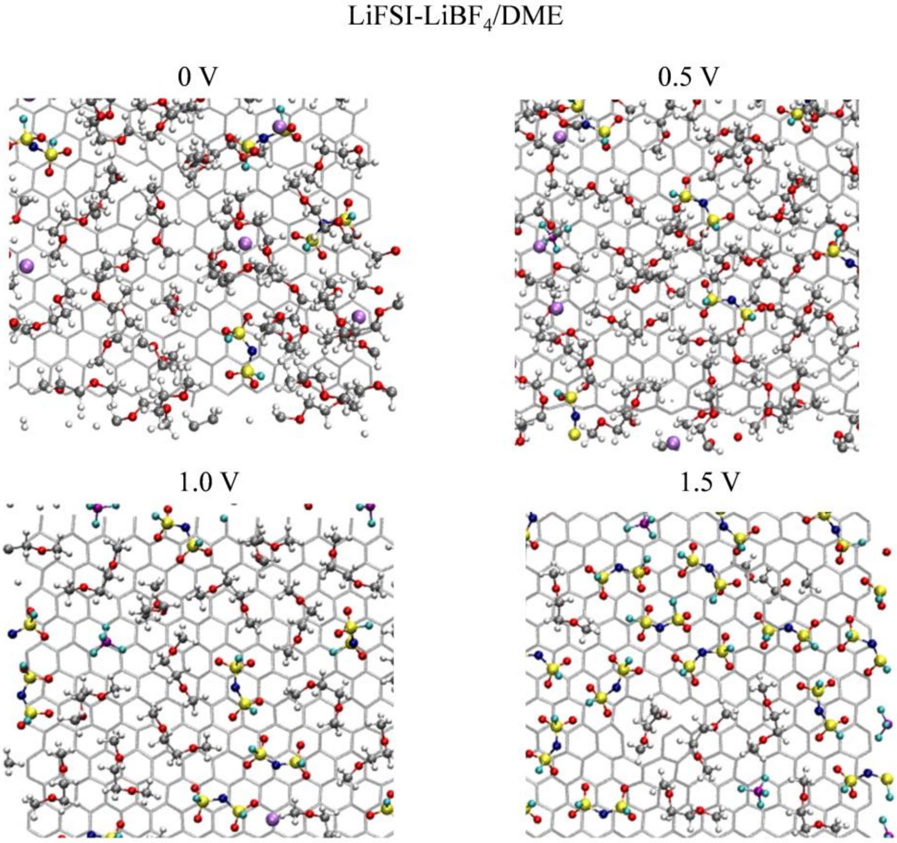

Supplementary Fig. 29. Local structure evolution of inner-Helmholtz interfacial regions at cathode surface in 1 M LiFSI-$LiNO_3$/DME under different voltage.

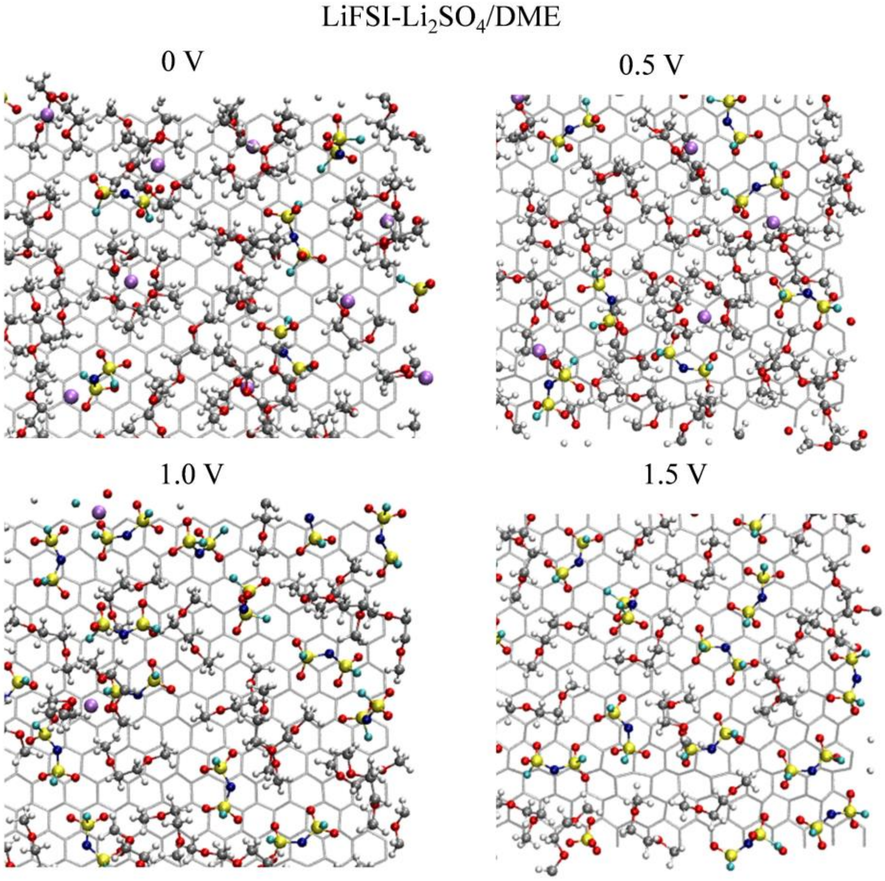

Supplementary Fig. 30. Snapshots of inner-Helmholtz interfacial regions of the cathode surface in 1 M LiFSI- Li_2SO_4/DME at different voltage.

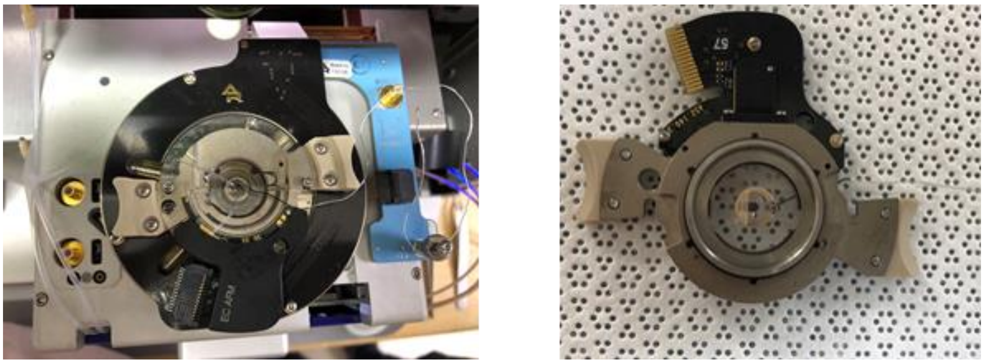

Supplementary Fig. 31. Photos of the liquid cup, a key part of the sealed AFM electrochemical cell.

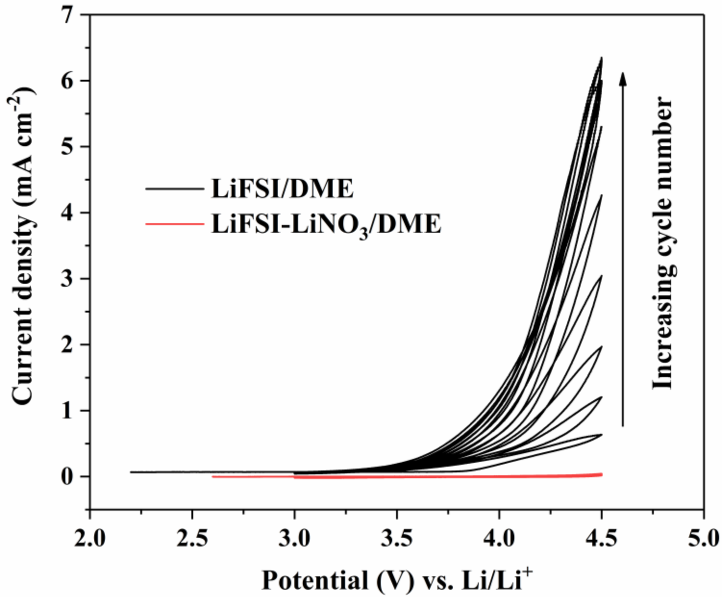

Supplementary Fig. 32. Cyclic voltammograms of LiFSI/DME and LiFSI-$LiNO_3$/DME using stainless steel working electrode in a 2032-coin cell setup. (Working electrode: stainless steel, counter and reference electrode: Li, scan rate: 10 mV/s, Voltage range: 3-4.5V).

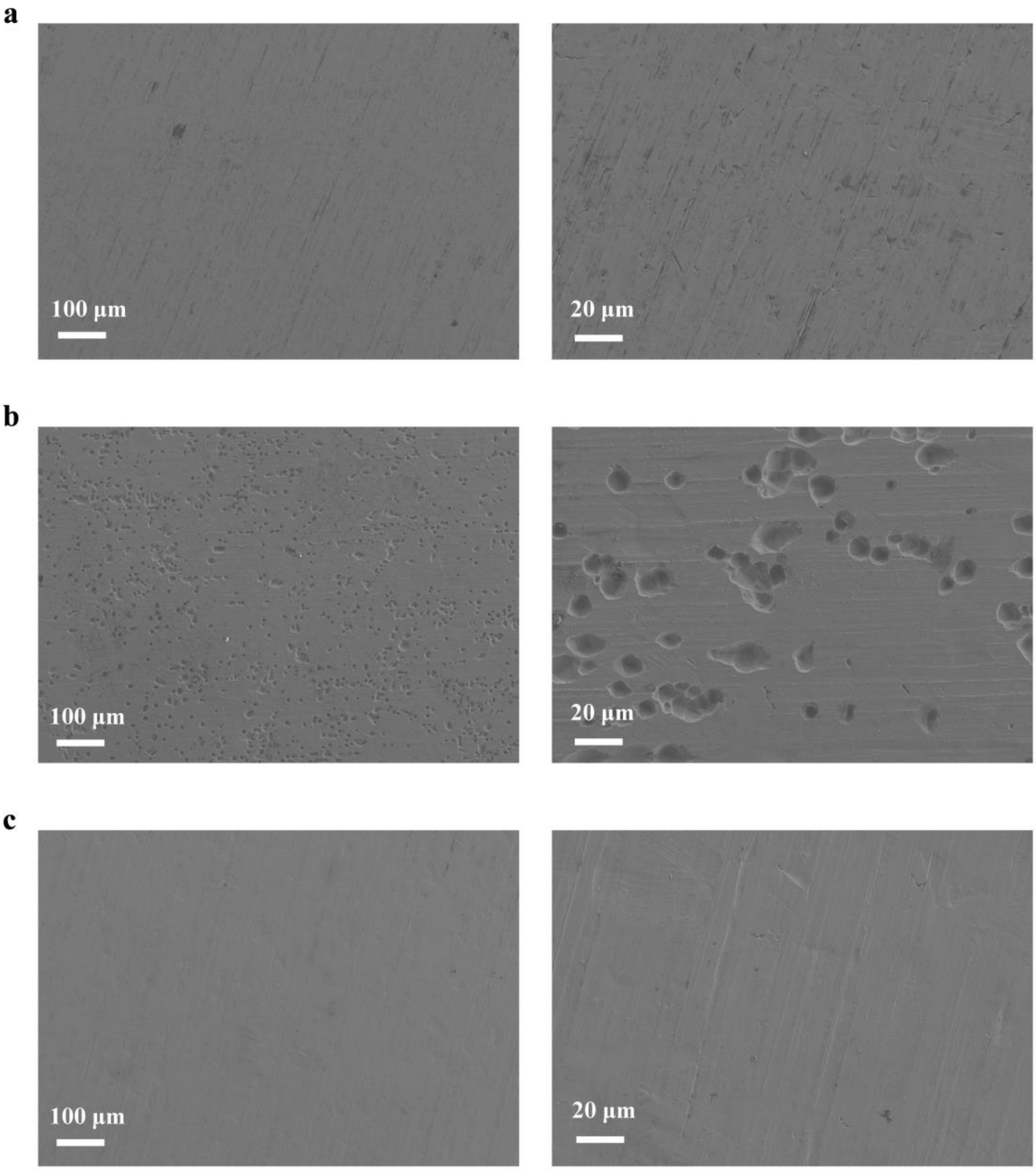

Supplementary Fig. 33. SEM images of (a) pristine stainless steel electrode and stainless steel electrode after Cyclic voltammograms test in (b) LiFSI/DME (c) LiFSI-$LiNO_3$/DME

Supplementary Table 1. The volume of the various anions calculated by DFT.

Anion	Occupied volume (Å^3)
TFO^-	90.07
$TFSI^-$	165.9
ClO_4^-	60.92
BF_4^-	49.77
PF_6^-	83.39
FSI^-	108.35
CO_3^{2-}	46.56
SO_4^{2-}	62.7
PO_4^{3-}	64.27
F^-	13.65
Cl^-	22.17
Br^-	26.63
I^-	32.4
$DFOB^-$	90.84
OA^-	67.91
NO_3^-	45.01

Supplementary Table 2. Li^+ transference number (t_{Li+}) computed from DC polarization measurements at 10 mV using the Bruce–Vincent method.

**Electrolytes**	**t (Li^+)**
$LiPF_6$/EC+DEC	0.31
LiFSI/DME	0.45
LiFSI-$LiNO_3$/DME	0.43
LiFSI/THF	0.49
LiFSI-$LiNO_3$/THF	0.46

Supplementary Table 3. Metrics of interest for previously published low temperature Li metal batteries.

	Cut-off voltage	Lowest operating temperature	Fast charging performance	Low-temperature charging	Low-temperature discharging	Low-temperature Capacity retention
Fan et al.	4.3 V	-85 ºC	/	×	√	56% at -85 ºC
Holoubek et al.	3.5 V	-60 ºC	/	√	√	76% at -60 ºC
Rustomji et al.	4.1 V	-60 ºC	/	×	√	61% at -60ºC
Dong et al.	3.0 V	-70 ºC	53% at 10 C	√	√	68% at -70 ºC
This work	4.5 V	-91 ºC	69.7% at 10 C	√	√	75% at -67 ºc<br>56% at -82 ºc<br>43% at -91 ºc

Supplementary Notes

Supplementary Note 1. Composition of Li metal performance of the LiFSI/DME and LiFSI-$LiNO_3$/DME

It is well known that the ether-based electrolyte is beneficial to enhance cycling of Li metal anode compared to carbonate electrolytes[1, 2]. Here, we found that the $LiNO_3$ additive could make the electrochemical behavior of Li metal anode even better. Although the coulombic efficiency of the bare DME electrolytes (1 M LiFSI in DME) is as high as 98.5% measured via the method proposed by Adams et al[3] (Supplementary Fig. 6a), the performance of Li||Cu half-cell is relatively unstable at long-term cycling (Supplementary Fig. 6b), and Li||Li cell is short-circuited only after 500 h's operation at a current density of 1 mA cm^{-2} (Supplementary Fig. 6c). In contrast, after adding $LiNO_3$, the Li||Cu half-cell exhibits improved coulombic efficiency (99.1%) and long-term cycling stability with an average CE of 98.86% for 150 cycles, and the assembled Li||Li symmetric cell can stably cycle for up to 1500 h at a current density of 1 mA cm^{-2}. SEM was further employed to observe the morphology of lithium metal deposited in different electrolytes. Interestingly, $LiNO_3$ induces Li deposition to grow into a more compact large Li chunks, which is in great contrast to the porous morphology of Li metal deposited from bare DME electrolyte (Supplementary Fig. 6d). This dendrite-free surface morphology is considered to be a key factor for high Coulombic efficiency. It has been reported that $LiNO_3$ can participate in the formation of SEI film on the surface of Li metal anode, and the resulting N-rich SEI layer can effectively inhibit the growth of lithium dendrite, thus improving the coulombic efficiency and long cycle life

of lithium metal anode[4].

Supplementary Note 2. Comparing the corrosion behavior of stainless steels in LiFSI/DME and LiFSI-LiNO$_3$/DME

For the LiFSI/DME system, a strong side reaction current value was observed in the cyclic voltammetry test when stainless steel was used as the working electrode (Supplementary Fig. 32). Such a strong Faradic current response is not only derived from the decomposition of the electrolyte but also the contribution of the aluminum corrosion reaction, as we found dense corrosion holes in the SEM photo of the recycled stainless steel (Supplementary Fig. 33b). In contrast, no apparent response current in the LiFSI/DME system was present within the voltage range of 3-4.5V (Supplementary Fig. 32), and the cycled stainless steel was as smooth as initially without corrosion pits (Supplementary Fig. 33c). It is well known that the problem of aluminum corrosion in high-concentration electrolyte systems can be ignored, so we believe that the constructed Li^+-rich EDL would not only protect DME from degradation, but also significantly inhibit the corrosion of stainless steel by FSI^-.

Supplementary References

1. Y. Jie, X. Ren, R. Cao, W. Cai, S. Jiao, Advanced Liquid Electrolytes for Rechargeable Li Metal Batteries. *Advanced Functional Materials* **30**, 1910777 (2020).
2. Y. Zhang *et al.*, Towards better Li metal anodes: Challenges and strategies. *Materials Today* **33**, 56-74 (2020).
3. B. D. Adams, J. Zheng, X. Ren, W. Xu, J.-G. Zhang, Accurate Determination of Coulombic Efficiency for Lithium Metal Anodes and Lithium Metal Batteries. *Advanced Energy Materials* **8**, 1702097 (2018).
4. X.-R. Chen *et al.*, A Diffusion-Reaction Competition Mechanism to Tailor Lithium Deposition for Lithium-Metal Batteries. *Angewandte Chemie-International Edition* **59**, 7743-7747 (2020).
